# Identification of glucocorticoid receptors as potential modulators of parasympathetic and sympathetic neurons within rat intracardiac ganglia

**DOI:** 10.3389/fnana.2022.902738

**Published:** 2022-09-23

**Authors:** Shaaban A. Mousa, Lukas Dehe, Noureddin Aboryag, Mohammed Shaqura, Antje Beyer, Michael Schäfer, Sascha Treskatsch

**Affiliations:** ^1^Department of Anaesthesiology and Intensive Care Medicine, Charité – University Berlin, Corporate Member of Freie Universität Berlin, Humboldt-Universität zu Berlin, Berlin Institute of Health, Campus Benjamin Franklin, Berlin, Germany; ^2^Department of Anaesthesiology, Ludwig-Maximilians-University Munich, Munich, Germany

**Keywords:** intracardiac ganglia, corticosteroid receptors, parasympathetic, sympathetic, sensory nerves

## Abstract

**Background:**

Emerging evidences indicate that glucocorticoid receptors (GR) play a regulatory role in cardiac function, particularly with regard to the autonomic nervous system. Therefore, this study aimed to demonstrate the expression and the precise anatomical location of GR in relation to the parasympathetic and sympathetic innervations of the heart.

**Methods:**

The present study used tissue samples from rat heart atria to perform conventional reverse-transcriptase polymerase chain reaction (RT-PCR), Western blot, and double immunofluorescence confocal analysis of GR with the neuronal markers vesicular acetylcholine transporter (VAChT), tyrosine hydroxylase (TH), calcitonin gene-related peptide (CGRP) as well as the mineralocorticoid receptor (MR).

**Results:**

Double immunofluorescence labeling revealed that GRs were co-expressed with VAChT in parasympathetic principal neuronal somata and nerve terminals innervating atrium. Also, GR colocalized with the sympathetic neuronal marker TH in a cluster of small intensely fluorescent (SIF) cells, on intracardiac nerve terminals and in the atrial myocardium. GR immunoreactivity was scarcely identified on CGRP-immunoreactive sensory nerve terminals. Approximately 20% of GR immunoreactive neuronal somata co-localized with MR. Finally, conventional RT-PCR and Western blot confirmed the presence of GR and MR in rat heart atria.

**Conclusion:**

This study provides evidence for the existence of GR predominantly on cardiac parasympathetic neurons and TH-immunoreactive SIF cells suggesting a functional role of cardiac GR on cardiovascular function by modulation of the cardiac autonomic nervous system.

## Introduction

It is well known that the major physiological adrenocorticosteroid hormones, glucocorticoids and mineralocorticoids, have a vital regulatory role in normal cardiovascular function (Schäffer et al., 2009; Rog-Zielinska et al., 2013; Gray et al., 2017). Indeed, the activation of GR and MR in the heart has a strong influence on cardiac development, physiology and pathophysiology (Oakley and Cidlowski, 2015; Richardson et al., 2016; Fernández-Ruiz, 2022). Recently, mice lacking both GR and MR in cardiomyocytes were resistant to the cardiac disease (heart failure) that develops in mice lacking myocardial GR only indicating the importance of a physiologic balance between these two receptor signaling pathways which seem to be vital for maintaining the healthy heart (Cruz-Topete et al., 2019; Oakley et al., 2019). Moreover, GR and MR are implicated in the development of cardiovascular remodeling during cardiac fibrosis and heart failure (HF) (Brilla and Weber, 1992; Weber, 2001; Cruz-Topete et al., 2019; Pianca et al., 2022).

Interestingly, antenatal administration of glucocorticoids enhances the sympathetic response after premature delivery and may be one mechanism by which the mother steroid release improves postnatal cardiovascular homeostasis (Segar et al., 2001). Moreover, the cardiac autonomic system has been shown to be sensitive toward mother glucocorticoids as reflected by the alteration of fetal heart rate variations in human fetuses (Dawes et al., 1994; Mulder et al., 2004). Accordingly, cardiac autonomic balance was preserved in neonates after a single course of antenatal betamethasone treatment (Schäffer et al., 2009).

It is well established that glucocorticoid corticosterone treatment modulates the baroreflex control of renal sympathetic nerve activity in rats (Scheuer and Mifflin, 2001). In the heart, the glucocorticoid corticosterone also modulates the baroreceptor reflex control of the heart rate in conscious rats (Scheuer and Bechtold, 2002). Consistently, Ives and Bertke (2017) reported that nearly half of cultured sympathetic superior cervical ganglion neurons expressed GR. The growth of sympathetically innervated myocardium was enhanced after glucocorticoid treatment, however, abolished after denervation (Torres and Tucker, 1995). Moreover, the exogenously administered glucocorticoids inhibited sympathetic outflow and interfered with the function of presynaptic alpha 2-adrenoceptors (Pacak et al., 1992; Lenders et al., 1995). Finally, Angelini et al. (2010) confirmed the activation of the cholinergic parasympathetic pathway during dexamethasone-induced insulin resistance and hyperinsulinemia in rats.

The heart has an intrinsic cardiac nervous system from the parasympathetic and the sympathetic divisions including intrinsic cardiac ganglia within atria (Mousa et al., 2010; Durães Campos et al., 2018). The intracardiac ganglia play an essential role on the heart rate, atrioventricular conduction, and myocardial contraction (Adams and Cuevas, 2004). Intriguingly, until now, the GR expression and the exact localization of GR with respect to the parasympathetic, sympathetic and sensory innervations of the heart remains elusive. Extending our previous study (Dehe et al., 2021), we set out to identify the expression of GR in the rat heart atria containing intracardiac ganglia using RT-PCR and Western blot. We further investigated the colocalization of GR with specific neuronal markers for parasympathetic neurons (vesicular acetylcholine transporter–VAChT), catecholaminergic (including sympathetic) neurons (tyrosine hydroxylase–TH), and primary afferent C-fibers (peptidergic) (CGRP) as well as MR by double immunofluorescence confocal microscopy. The results of these findings may provide morphological evidence for a possible corticosteroid control of the cardiac autonomic innervation.

## Materials and methods

### Animals

In order to avoid potential interferences of menstrual and hormonal fluctuations and for better comparisons with previous studies that were done in male rats only, we conducted our experiments in naïve adult male Wistar rats (200–250 g) (breeding facility, Charité-Universitätsmedizin Berlin, Germany) after approval by the local animal care committee (reference# G0024/14, Landesamt für Gesundheit und Soziales, LaGeSo Berlin). Animal care and experiments were performed in accordance with the European Directive introducing new animal welfare and care guidelines (2010/63/EU) as well as with the ARRIVE guidelines.^1^

### Tissue preparation

Rats were deeply anesthetized with isoflurane and the heart tissue—including the right and left atria, the left precaval vein, short lengths of the pulmonary veins and the superior and inferior vena cava—were removed from adult rats for RT-PCR and Western blot.

### Conventional reverse-transcriptase polymerase chain reaction

Conventional reverse-transcriptase polymerase chain reaction (RT-PCR) analysis was used to confirm the presence of GR and MR specific mRNA within rat atria as described previously (Mousa et al., 2010; Tafelski et al., 2019). Tissues of the atria from 5 rats were collected in lysis-buffer. RNA was extracted using a Qiagen Mini Kit (Qiagen, Hilden, Germany) for each experiment. One microliter oligo dT was added to 10.4 μl RNA, incubated at 25°C for 10 min, then at 42°C for 60 min, finally at 99°C for 5 min and transferred onto ice. cDNA was stored at –20°C. The following specific primers were used for GR: forward primer: 5′-CATCTTCAGAACAGCAAAATCGA-3′, reverse primer: 5′-AGGTGCTTTG GTCTGTGGG ATA-3′ (Ensembl, Accession Nr: NM_012576.2); for MR: rorward primer; 5′-CCAAGGTACTTCCAGGATTTAAAAAC-3′, reverse primer; 5′-AACGATGATAGACAC ATCCAAGAATACT-3′ (Ensemble Accession No: NM_013131.1). For RT-PCR analysis, Maxima Hotstart Green Enzyme (Thermo Fisher Scientific, Waltham, MA, USA) was used for the subsequent steps. Amplification was carried out for 40 cycles, each consisting of 30 s at 95°C; for GR, MR and 18S cycles consisted of 30 s at 60°C. PCR products were run on a 2% NuSieve^§^ agarose gel with a 100 bp marker (GeneRuler DNA Ladder, ThermoFisher Scientific, Waltham, MA, USA) and stained with ethidium bromide. Specific bands were visualized on an agarose gel by a gel documentation system (EasyDoc, Fa. Bio-Rad Laboratories GmbH, Feldkirchen, Germany). RT-PCRs were controlled by a reverse transcriptase-free and a RNA-free negative control.

### Western blot

Tissues of the atria and dorsal root ganglia (DRG, only for supplemental data in Supplementary Figure 3) from 5 rats were solubilized and then Western blot analysis was performed as previously described (Mousa et al., 2010; Li et al., 2018; Mohamed et al., 2020). After blotting, the membranes were blocked in 3% BSA for 2 h and incubated with rabbit anti-GR (a gift from M. Kawata, Kyoto Prefectural University of Medicine, Japan; 1:4,000 in 3% BSA), mouse anti-MR (private gift from Prof. Elise Gomez-Sanchez, Jackson, USA; 1:200 in 3% BSA) (Li et al., 2018) overnight at 4°C. A molecular weight marker (ladder) was used from Precision Plus Protein Dual Color Standards, (Bio-Rad Laboratories GmbH, Feldkirchen, Germany). The GR antibody (M. Kawata) has previously been shown in COS-1 cells with or without GR transfection to be highly specific (Han et al., 2005). Also, the MR antibody (E. Gomez-Sanchez) has previously been shown in heart tissue to be highly specific (Gomez-Sanchez et al., 2006, 2011). After incubation with the secondary antibody (peroxidase-conjugated goat anti-rabbit, 1:20,000, rabbit anti-mouse 1:5,000, Jackson ImmunoResearch, West Grove, PA, USA) for 2 h at room temperature, reactive protein bands were digitally visualized using ECL solutions (SuperSignal West Pico, Thermo Scientific, Waltham, MA, USA) in ChemiDoc MP Imager (BioRad Laboratories GmbH, Feldkirchen, Germany). A negative control was obtained incubating the filter with a solution in which the primary antibody was replaced by BSA.

### Immunohistochemistry

#### Tissue preparation

Adult rats (*n* = 5) were deeply anesthetized with isoflurane and transcardially perfused with 100 ml warm saline solution, followed by 300 ml 4% (w/v) paraformaldehyde (Sigma, St. Louis, Taufkirchen, Germany) in 0.16 M phosphate buffer solution (PBS) (pH 7.4) as previously described (Mousa et al., 2010). After perfusion the ventricles were removed by cutting along the atrioventricular groove and the aorta and pulmonary trunk were gently detached. The remaining tissue included the right and left atria, the left precaval vein, short lengths of the pulmonary veins and superior and inferior vena cava were now also removed and fixed in the same fixative for 90 min, and then cryoprotected overnight at 4°C in PBS containing 10% sucrose. The tissues were then embedded in Tissue-Tek compound (OCT, Miles Inc. Elkhart, IN) and frozen. The tissues were cut tangentially to the atrial wall beginning at the most superior aspect of the atria and ending in the ventricular myocardium at the superior aspect of the right and left bundle branches into 50-μm-thick sections in a cryostat. The sections were collected in PBS (floating sections). In addition, 10-μm-thick sections were mounted on gelatin coated slide.

#### Double immunofluorescence staining

Double immunofluorescence staining was processed as described previously (Mousa et al., 2007). Floating tissue sections or slide mounted tissue sections were incubated for 60 min in PBS containing 0.3% Triton X-100 (Sigma, St. Louis, Taufkirchen, Germany), 1% BSA, 10% goat serum (Vector Laboratories, CA, USA) (blocking solution) to prevent non-specific binding. Tissue sections were then incubated overnight with the following primary antibodies: (1) polyclonal rabbit anti-GR (private gift from M. Kawata, Koyoto, Japan (this antibody has previously been shown in COS-1 cells with or without GR transfection to be highly specific and displays similar specific staining on dorsal root ganglion neurons with a different commercial GR antibody from Santa Cruz as previously described in Shaqura et al. (2016a). In combination with polyclonal goat anti-VAChT, polyclonal guinea pig anti-CGRP (Peninsula Laboratories, 1:1000), monoclonal mouse anti-TH (Immunostar Inc., WI, USA, 1:2,000) or the monoclonal mouse anti-MR (private gift from Celso E. Gomez-Sanchez, University of Mississippi, Jackson, Mississippi) which has previously been shown in heart tissue to be highly specific (Gomez-Sanchez et al., 2006, 2011).

After incubation with primary antibodies, the tissue sections were washed with PBS and then incubated with Alexa Fluor 594 donkey anti-rabbit antibody (Vector Laboratories, Newark, CA) in combination with Alexa Fluor 488 goat anti-guinea pig, anti-mouse, anti-chicken or anti-sheep antibody (Invitrogen, Waltham, MA, USA). Thereafter, sections were washed with PBS, and the nuclei-stained bright blue with 4’-6-Diamidino-2-phenylindole (DAPI) (0.1 μg/ml in PBS) (Sigma, St. Louis, MO). Finally, the tissues were washed in PBS, mounted in Vectashield (Vector Laboratories, Newark, CA) and imaged on a confocal laser scanning microscope, LSM510, equipped with an argon laser (458/488/514 nm), a green helium/neon laser (543 nm), and a red helium/neon laser (633 nm; Carl Zeiss, Göttingen, Germany) as described previously (Li et al., 2018). To demonstrate specificity of staining, the following controls were included as described in our previous studies (Li et al., 2018): omission of either the primary antisera or the secondary antibodies. Single optical slice images were taken using × 20 Plan-Neofluar air interface or × 40 Plan-Neofluar oil interface objective lens with a confocal laser scanning microscope, LSM510 (633 nm; Carl Zeiss, Göttingen, Germany).

#### Quantification of immunostaining

Imaged on a confocal laser scanning microscope, LSM510, equipped with an argon laser (458/488/514 nm), a green helium/neon laser (543 nm), and a red helium/neon laser (633 nm; Carl Zeiss, Göttingen, Germany). Single optical slice images were taken using ×10 or ×20 Plan-Neofluar air interface or ×40 Plan-Neofluar oil interface objective lens. The settings of the confocal microscope were established using a control section and kept unchanged for all subsequent acquisitions. For the quantitative evaluation of all immunofluorescence double staining, a two-color overlay image analysis given by the image analysis program ImageJ^®^ (Version 1.4.1) was applied^2^ as previously described (Shimojo et al., 1997; Schneider et al., 2012). In more detail, the different color channels, each identifying distinct target structures, were separated by using the plug-in (color deconvolution), thus the color signal can quantitatively be evaluated. A manually specified area was identified for each specifically colored area. Intensity thresholds were assigned, so that a maximum degree of integrated area of stained target structure was identified, while minimizing possible background activities. Areas above the threshold value were defined as positive and indicated information about the percentage of the immunostained area in relation to the previously selected total area. Values below the threshold were eliminated as background. The threshold value was kept constant for all sections. With the help of ImageJ, the parameter percentage area (% stained area) was calculated using the software. The percentage area was defined as the specific-colored area in relation to the total area of a photographed tissue preparation. All calculated quantitative color intensities are presented as% immunoreactive area in the manuscript (see also Supplementary Figure 1). Data were presented as median plus range. Data were obtained from a minimum of 3–4 sections per rat and a total of five rats.

## Results

### Localization of glucocorticoid receptors in cardiac parasympathetic vesicular acetylcholine transporter-immunoreactive neurons within rat atria

**FIGURE 1:**
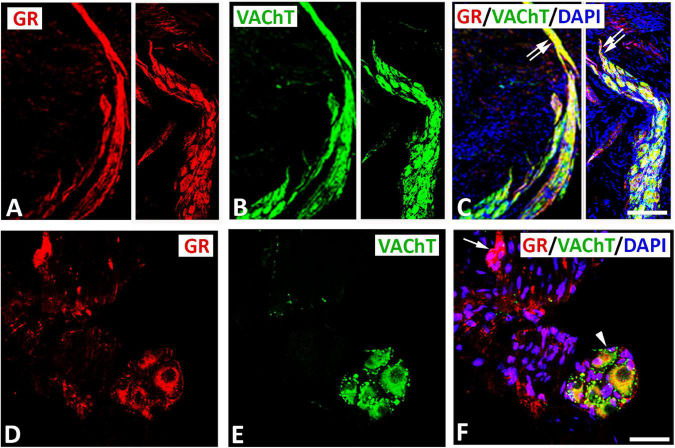
Confocal microscopy of GR **(A,D)** (*red fluorescence*) and parasympathetic neuronal marker VAChT **(B,F)** (*green fluorescence*) double immunofluorescence in intracardiac ganglia **(A–F)**. **(A–C)** (**right** and **left**) Show GR co-expressed with VAChT immunoreactivity (*yellow fluorescence*) in a cluster of neuronal soma. Note, VAChT-immmunoreactive bundles of nerve fibers extending from ganglion neuronal somata. **(D–F)** Show large size principal neuronal somata in rat atria coexpressing GR and VAChT (arrowhead) in close proximity with a cluster of 5 small size neurons (as indicated with five nuclei) which express GR only (arrow) and lack VAChT immunoresctivity. **(D,F)** Show DAPI nuclear staining (*bright blue*). Bar: **(A–C)** = 40 μm; **(D–F)** = 120 μm.

**FIGURE 2:**
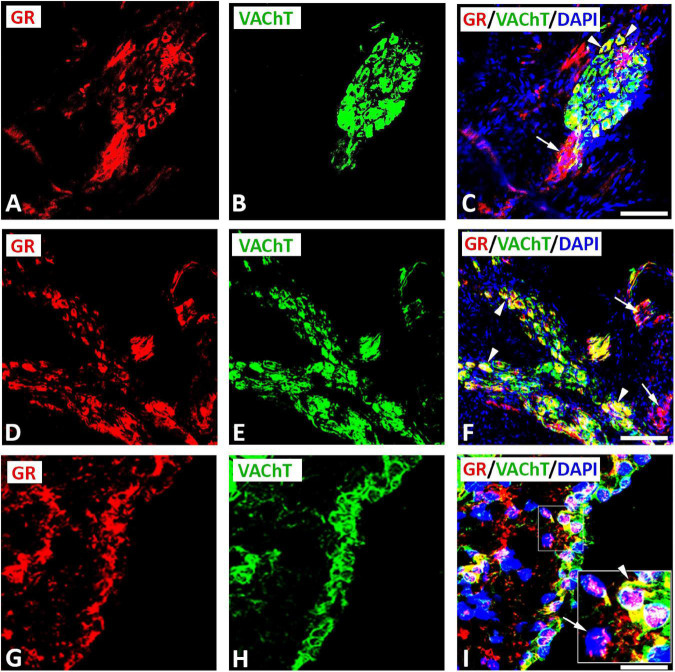
Confocal microscopy of GR **(A,D,G)** (*red fluorescence*) and parasympathetic neuronal marker VAChT **(B,E,H)** (*green fluorescence*) double immunofluorescence in intracardiac ganglia **(A–I)**. **(A–F)** Show GR co-expressed with VAChT (*yellow fluorescence*) in large diameter neuronal soma (arrowhead), however, cluster of small diameter neuronal somata expressed GR only (arrow). **(G–I)** Higher magnification of gelatin-embedded transverse sections of atria (10μm thickness). Note a clear localization of GR immunoreactivity with VAChT-immunoreactive neuronal soma; however, some neurons expressed GR or VAChT only. The insert in **(I)** represents a higher magnification of a selected area showing GR expression in a nucleus (arrowhead) or at the periphery of neuron (arrow). **(C,F,I)** Show DAPI nuclear staining (*bright blue*). Bar: **(A–I)** = 60 μm; a higher magnification of **(I)** = 120 μm.

**FIGURE 3:**
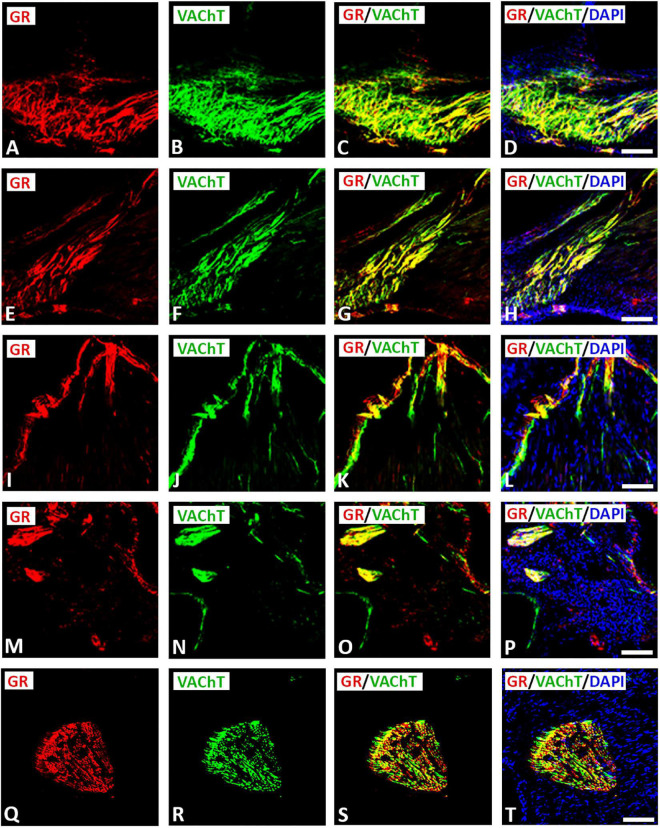
Double immunofluorescence confocal microscopy of nerve terminals within rat atria co-expressing GR **(A,E,I,M,Q)** (*red fluorescence*) and/or parasympathetic neuronal marker VAChT **(B,F,J,N,R)** (*green fluorescence*). **(A–H)** Show predominant localization of GR with a VAChT-immunoreactive network of nerve fibers within rat atria. **(I–P)** Show bundles of VAChT-positive nerve fibers co-expressing GR immunoreactivity (*yellow fluorescence*) within rat atrium. **(Q–T)** Represent cross-sectioned nerves within heart atria contain high populations of VAChT-immunoreactive fibers expressing GR immunoreactivity (*yellow fluorescence*). **(D,H,L,T,P)** Show DAPI nuclear staining (*bright blue*). Bar: **(A–P)** = 40 μm; **(Q–T)** = 60 μm.

Double immunofluorescence labeling for confocal microscopy showed that GR immunoreactivity was detectable in large diameter parasympathetic neuronal somata, i.e., VAChT-immunoreactive principal parasympathetic neurons of intracardiac autonomic ganglia (Figures 1, 2). Some GR-immunoreactive neurons lacked VAChT immunoreactivity and vice versa (Figures 1A–F, 2A–I). In addition, clusters of small size neuronal cells—most likely SIF cells—expressing GR only and lacking VAChT immunoreactivity can be observed and are located in close proximity of (spheroid in shape) VAChT-immunoreactive principal parasympathetic neurons (Figures 1D–F, 2A–F). In tissue sections of rat atria, quantification of the median[range]% values of the area containing intracardiac ganglia exhibited GR colocalizing with VAChT (yellow fluorescence) of up to 42[10–68]% overlap, whereas 48% did not colocalize (red fluorescence only). Moreover, 48[12–71]% of VAChT immunoreactive area colocalized with GR (yellow fluorescence), whereas 52% did not (green fluorescence only). Additionally, a thick bundle of vagal afferent endings consisting of VAChT-positive fibers co-expressed GR and projected toward intracardiac ganglia to make some contact to neuronal somata (Figures 1A–C). In addition, GR immunoreactivity was demonstrated mostly in networks of VAChT- immunoreactive nerve fibers (Figures 3A–H) as well as in bundles (Figures 3I–P) of nerve fibers intensively innervating rat heart atria (Figure 3). Also, epicardial nerves coexpressing GR and VAChT were detected in rat atria (Figures 3Q–T). Some VAChT-immunoreactive fibers lacked GR immunoreactivity and vice versa.

### Localization of glucocorticoid receptors in catecholaminergic (including sympathetic) tyrosine hydroxylase-immunoreactive neurons within rat atria

**FIGURE 4:**
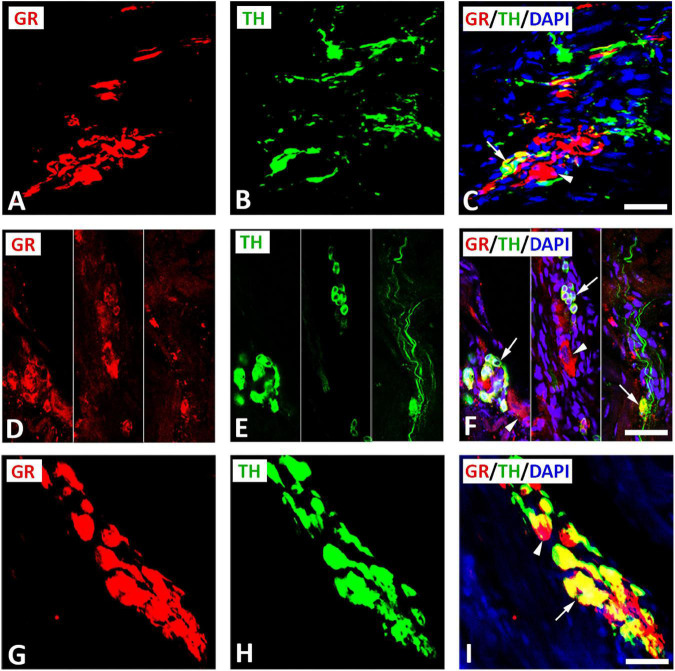
Confocal microscopy of GR **(A,D,G**) (*red fluorescence*) and sympathetic neuronal marker tyrosine hydoxylase (TH) **(B,E,H)** (*green fluorescence*) double immunofluorescence in intracardiac ganglia **(A–I)**. **(A–C)** Show GR co-expressed with TH (*yellow fluorescence*) in cluster of three small intensely fluorescent (SIF) cell (arrow), however, some neuronal soma express GR in large size principal cell (arrowhead) or TH only. **(D–F)** (**right** and **middle**) Show a cluster of TH-immunoreactive small intensely fluorescent (SIF) cells (arrow) co-express GR (*yellow fluorescence*) that were much smaller than the surrounding large size principal ganglion cells (*red fluorescence*) (arrowhead). **(D–F)** **(Left)** show unipolar small intensely fluorescent SIF cells expressing GR (arrow). **(G–I)** Show a cluster of small cells of cardiac ganglia coexpressing GR (arrow) and TH, however some cell express GR only (arrowhead). **(C,F,I)** Show DAPI nuclear staining (*bright blue*). Bar: **(A–F)** = 40 μm; **(G–I)** = 80 μm.

Double immunofluorescence staining for confocal microscopy identified GR immunoreactivity with the catecholaminergic neuronal marker TH on clusters of small intensely fluorescent (SIF) cell-like neurons of intracardiac ganglia (Figures 4A–I) consistent with previous descriptions of SIF cells by McMahan and Purves (1976), Heym et al. (1994), and Mousa et al., 2010, 2011. Apart from this colocalization, some cells, which immunostained for TH, lacked GR immunoreactivity and vice versa (Figure 4). SIF cells were distinguished from large-diameter principal neurons that were immunoreactive for GR only (see Figures 4A–F), but did not co-localize with TH. Clusters of SIF cells colocalizing with GR were commonly located in close vicinity of large size principal neuronal somata (see Figures 4A–F). In addition, there are unipolar small intensely SIF cells expressing GR (Figures 4D–F). Also, intracardiac ganglia were innervated by preganglionic fibers positive for TH (Figures 4A–C). In tissue sections of rat atria, quantification of the median [range]% values of the area of GR colocalizing with TH (yellow fluorescence) revealed up to 45[18–70]% overlap, whereas 55% did not (red fluorescence only) (Figures 3A–I). Moreover, 38[19–79]% of TH immunoreactive area colocalized with GR (yellow fluorescence), whereas 62% did not (green fluorescence only).

**FIGURE 5:**
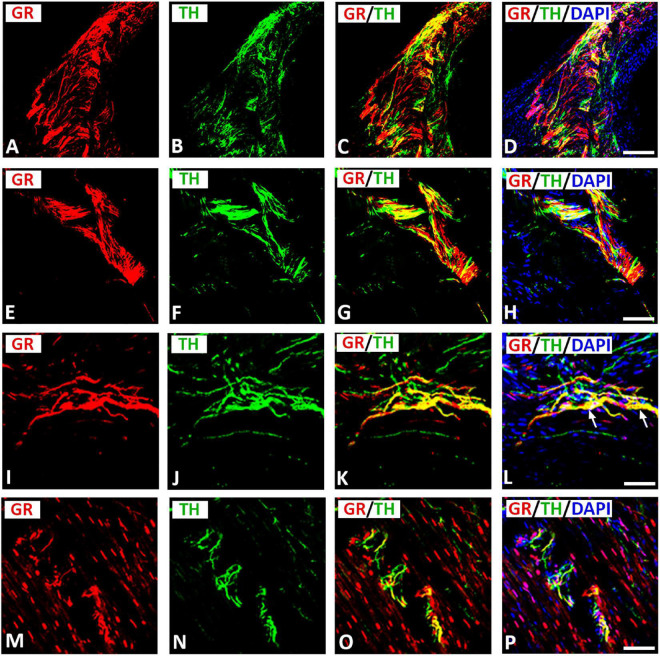
Confocal microscopy of GR **(A,E,I,M)** (*red fluorescence*) and sympathetic neuronal marker tyrosine hydoxylase (TH) (**B,F,J,N**) (*green fluorescence*) double immunofluorescence of nerve terminals within atria (I-P). **(A–D)** Show colocalization (*yellow fluorescence*) of GR with TH-immunoreactive network of nerve fibers within rat atria, however some nerve fibers express only GR or TH. **(E–L)** Show GR expressed in TH-immunoreactive nerve bundles (arrow) or single axons of epicardial nerve within rat atria. **(M–P)** Show TH-positive nerve fibers running in small bundles within the myocardium coexpressing GR (*yellow fluorescence*). Note, many cardiomyocyte nuclei express GR. **(D,H,L,P)** Show DAPI nuclear staining (*bright blue*). **(A–P)** = 40 μm.

Moreover, GR immunoreactivity was colocalized on a network of TH-immunoreactive nerve fibers innervating rat heart atria (Figures 5A–D). Some TH immunoreactive fibers lacked GR immunoreactivity and vice versa. Apparently, GR immunoreactivity was more extensive in VAChT-immunoreactive than in TH-immunoreactive nerve terminals within rat atria (see Figures 3E–H). Also, epicardial fiber bundles consisting of TH-positive nerve fibers co-expressed GR densely innervating rat atria (Figures 5E–L). Some terminal and pre-terminal axons colocalizing GR with TH were observed throughout the atrial myocardium (Figures 5M–P). Additionally, GR was expressed in atrial myocardium and many cardiomyocyte nuclei (Figures 5M–P).

### Coexpression of glucocorticoid receptors with mineralocorticoid receptor in cardiac neurons within rat atria

**FIGURE 6:**
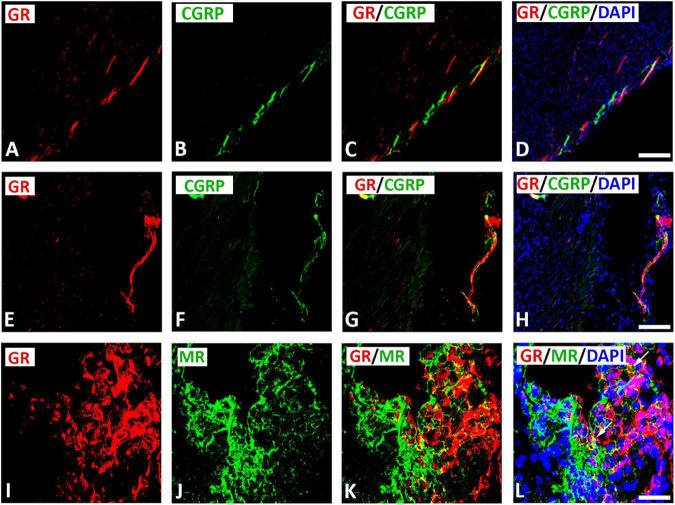
Confocal microscopy of GR **(A,E,I,M)** with sensory neuronal marker CGRP **(B,F)** or MR **(J,N)** immunofluorescence (*red fluorescence*) in intracardiac ganglia of tissue sections. **(A–H)** Show GR-IR nerve fibers rarely express CGRP within atrium tissue sections. **(I–L)** Is gelatine mounted tissue section of atria (10 μm thickness) showing some populations of neuronal cells co-expressing GR with MR (*yellow fluorescence*) (arrow); however, other populations of neuronal cells expressed GR or MR alone. In addition, neuronal cells express only GR are in close contact with MR-immunoreactive cells. **(A–H)** = 40 μm; **(I–L)** = 120 μm.

Our immunofluorescence analysis showed that GR-immunoreactive nerve fibers rarely (9[2.5–24]%) expressed CGRP within atrium tissue (Figures 6A–H). However, double immunofluorescence confocal microscopy of rat atria sections showed that some populations of GR-immunoreactive neuronal somata co-expressed MR immunoreactivity up to 20[12–27]% (median[range]%) of immunoreactive area in intracardiac ganglia (Figures 6I–L). Moreover, 33[13–35]% of immunoreactive area of MR colocalized with GR (yellow fluorescence), whereas 67% did not (green fluorescence only).

### Identification of glucocorticoid receptors and mineralocorticoid receptor specific mRNA as well as their proteins in rat atria

Using different primer pairs, the expected GR and MR specific RT-PCR-products (Supplementary Figure 2A) were identified in atrial heart tissue of naïve rats using conventional RT-PCR. Following gel electrophoresis of the RT-PCR products, the expected 84-bp cDNA fragment for GR and the expected 85-bp cDNA fragment for MR (Supplementary Figure 2A) were detected.

Using specific antibodies for GR or MR, gel electrophoresis with subsequent immunoblots of tissue extracts from rat atria consistently showed the predicted GR and MR specific protein bands (Supplementary Figures 2B,C, 3) at the expected molecular weights of 87 and 102 kDa, respectively. These were consistent with GR and MR specific protein bands previously shown in rat spinal cord, DRG neurons and peripheral human skin (Shaqura et al., 2016a,b; Al-Madol et al., 2017; Li et al., 2018; Supplementary Figure 3).

## Discussion

Glucocorticoids are critical regulators of heart function, particularly with respect to the cardiac neuronal conduction system (Cruz-Topete et al., 2020), however, the precise allocation of its receptors within the intrinsic neuronal network of the heart remains poorly investigated. In line with earlier reports (Arriza et al., 1987; Reul et al., 1989; Dehe et al., 2021), we were able to identify GR and MR specific mRNA and corresponding receptor proteins in rat heart atria. Double immunofluorescence confocal microscopy demonstrated colocalization of GR with VAChT (42%) in large diameter parasympathetic principal neuronal soma and with networks of VAChT-immunoreactive nerve fibers innervating adult rat atria. In addition, GR was expressed in TH-immunoreactive (45%) SIF cells and on nearby TH-immunoreactive nerve terminals as well as in atrial myocardium. Finally, some populations of GR-immunoreactive neuronal soma coexpressed MR (20%) in intracardiac ganglia suggesting that GR and MR were mainly expressed in different neuronal populations. These findings may provide first morphological evidence of the expression of GR on different ganglia and neuronal populations of the intrinsic cardiac nervous system suggesting a possible local corticosteroid control of the autonomic heart innervation.

To investigate the exact anatomical localization and cell type of GR within rat atria, we performed double immunofluorescence confocal microscopy using specific markers for parasympathetic (VAChT), catecholaminergic (including sympathetic) (TH), and peptidergic neurons (CGRP). The present results revealed a colocalization of GR immunoreactivity with VAChT (42%) in cardiac large diameter parasympathetic principal neuronal soma and nerve fibers intensely innervating adult rat atria. Our morphological findings are in line with previous functional studies, e.g., by Segar et al. (2001) who found that GR activation by corticosterone resulted in the modulation of the baroreceptor reflex control of renal sympathetic nerve activity. Moreover, GR agonist dexamethasone increased the sympathetic and decreased the parasympathetic component of the heart rate variability resulting in an autonomic imbalance of the heart (Duchatsch et al., 2018). Also, the administration of pregnant mice with dexamethasone transiently altered diastolic function in the mouse fetal heart and indirectly supported the modulation of cardiac sympathetic and parasympathetic activity (Agnew et al., 2019). Indeed, antenatal glucocorticoid betamethasone administration maintained the balance of neonatal cardiac sympathetic and parasympathetic activity (Schäffer et al., 2009). Taken together, our findings support a functional role of GR in the modulation of the parasympathetic neuronal network within rat heart.

Regarding the sympathetic innervation of the rat heart, GR in our hands also colocalized with TH-immunoreactive SIF cells and sympathetic nerve fibers that course through intracardiac ganglia and between cardiomyocytes. These TH-immunoreactive SIF cells are considered to be postsynaptic sympathetic neurons or catecholaminergic paraneuronal cells in intrinsic ganglia (Adams and Cuevas, 2004). Our findings are consistent with the previous study by Ives and Bertke (Ives and Bertke, 2017) demonstrating that nearly half of cultured primary adult murine neurons from the sympathetic superior cervical ganglion expressed the GR, which makes them susceptible to stimulation by glucocorticoids. Consistently, Segar et al. (2001) suggested that modulation of the cardiovascular and autonomic function in glucocorticoid-treated premature lambs is dependent, in part, on a generalized sympatho-excitatory response. Also, glucocorticoids can modulate arterial baroreceptor reflex control of renal sympathetic nerve activity (Scheuer and Mifflin, 2001; Bechtold and Scheuer, 2006). The previous reports indicated that the glucocorticoid treatment of superior cervical ganglia of rats at birth increased the SIF cells 10 times compared to untreated siblings (Allison and Gough, 1985). Consistently, Doupe et al. (1985) reported that no SIF cells were observed after 3 weeks in culture in the absence of glucocorticoids confirming the vital requirement of GR on SIF cells. The present study showing localization of GR in intracardiac catecholaminergic SIF cells provides a novel understanding of the molecular basis of heart control through glucocorticoids.

In addition to parasympathetic neurons and catecholaminergic SIF cells, GR was also expressed in atrial myocardium (Figures 5M–P). Our confocal microscopy analysis confirmed previous evidence reviewed by Oakley and Cidlowski (2013) showing that GR was localized in cardiomyocytes, The deficiency in cardiomyocyte glucocorticoid signaling leads to spontaneous cardiac hypertrophy, heart failure, and death, revealing a mandatory role for GR in maintaining normal cardiovascular function (Oakley and Cidlowski, 2013). Moreover, cardiomyocyte-specific GR knockout in mice leads to irregular Ca^2+^ signaling, impaired contractility and heart failure (Oakley and Cidlowski, 2013). The growth of sympathetically innervated myocardium is enhanced with glucocorticoid exposure, but growth of non-innervated myocardium (e.g., fetal heart) may be compromised by excessive glucocorticoid exposure (Torres and Tucker, 1995).

In contrast to the expression of GR on sympathetic SIF cells and parasympathetic neurons, our results revealed only scarce co-localization of GR with CGRP-immunoreactive sensory nerve fibers throughout intracardiac ganglia of rat atrium. This finding is in line with the previous reports by Ives and Bertke (2017) in which corticosterone selectively modulates acute Herpes Simplex Virus 1 productive infections in murine sympathetic, but not sensory neurons.

In the distal nephrons of the kidney, MR is co-expressed with the closely related glucocorticoid receptor (GR) in epithelial cells (Farman et al., 1991; Farman and Rafestin-Oblin, 2001). Here in rat heart atria, double immunofluorescence confocal microscopy revealed only a minor colocalization of GR-immunoreactive neuronal soma with MR (20%) suggesting that GR and MR were mainly expressed in different neuronal populations.

Supporting our immunohistochemical demonstration of GR and MR in rat heart atria, we were able to isolate GR and MR specific transcripts in rat heart atria by conventional RT-PCR confirming the presence of GR and MR in rat heart atria. Similar to our previous studies in sensory dorsal root ganglia (Shaqura et al., 2016a,b; Li et al., 2018), the expected 84-bp fragment for GR and 85-bp fragment for MR were also identified in rat heart atria. Running Real-Time-PCR, the detection thresholds (Ct values) of the GR specific PCR cycle number were very similar in rat atria (21.9 ± 0.6), kidney (21.7 ± 0.8) or adrenal glands (21.8 ± 0.3) indicating no major discrepancies in the tissue specific expression levels (unpublished data).

These findings are consistent with earlier reports that detected predominantly MR mRNA in the rat heart by Northern blot analysis (Arriza et al., 1987; Reul et al., 1989). Moreover, MR as well as GR mRNA and proteins were detected in mice cardiomyocytes (Oakley and Cidlowski, 2013; Seidel et al., 2019). In support of these findings, our western blot experiments in rat atria recognized the predicted protein bands of approximately 87 and 102 kDa for GR and MR, respectively.

Several limitations should be addressed in order to stimulate further studying in the future. First, our experiments investigated only the expression of GR and MR within cardiac ganglia without any additional experiments exploring their genomic function in order to understand its response to pathological conditions. Second, we have conducted our investigation only in naïve male rats, however the future comparison between male and female rats as well as naïve and diseased rats (e.g., chronic-volume overload-initiated heart failure) established in our laboratory will strength our findings. Our experiments used only immunohistochemistry and conventional PCR, but radioimmunoassay binding and *in situ* hybridization will strength our findings. Finally, our study was conducted only in male Wistar rats without any reference to female rats.

In summary, our results substantiate evidence of the expression of GR transcripts and proteins in intracardiac ganglia within rat atria. Immunofluorescence confocal microscopy allowed the precise anatomical localization of GR within intracardiac ganglia. Consequently, we were able to colocalize GR with VAChT-immunoreactive of large diameter principal neurons, with TH in small TH-immunoreactive SIF cells, but rarely with CGRP in afferent nerve terminals within intracardiac ganglia and atrial myocardium, where they function as a potential target for a possible control of intrinsic cardiac sympathetic and parasympathetic divisions. Our findings can be seen as a step toward a better understanding of the neural control of the rat heart by the local intrinsic corticosteroid system which might imply a functional role under cardiac disease states. Our study stimulates further functional studies to confirm the possible modulatory role of corticosteroids in cardiac autonomic innervation.

## Data availability statement

The original contributions presented in this study are included in the article/Supplementary material, further inquiries can be directed to the corresponding author.

## Ethics statement

The animal study was reviewed and approved by reference# G0024/14, Landesamt für Gesundheit und Soziales, LaGeSo Berlin.

## Author contributions

SM, LD, ST, and MSc designed the experiments. SM, LD, NA, and MSh performed the experiments. SM, LD, NA, AB, MSc, and ST performed the analyses and interpretation of the experiments. SM, LD, MSc, AB, and ST wrote part of the manuscript. All authors reviewed the manuscript and approved the submitted version.

## Footnotes

1
https://arriveguidelines.org

2
https://imagej.nih.gov/ij/index.html

## Supplementary material

Supplementary Figure 1 Example of quantitative evaluation of immunohistochemical staining within rat atria using the version 1.41 of the image analysis program ImageJ^®^ (http://rsbweb.nih.gov/ij/). The additional use of the plug-in (color deconvolution) allowed the separation of the different color channels each identifying distinct target structures, whose color signal can, thus, be quantitatively evaluated. With the help of ImageJ, the parameter percentage area (% stained area) was calculated. The percentage area was defined as the specific-colored area in relation to the total area of a photographed tissue preparation.

Supplementary Figure 2 Detection of GR and MR mRNA **(A)** and proteins **(B)** by conventional RT-PCR and western blots, respectively, in rat atria containing intracardiac ganglia. **(A)** GR and MR mRNA were amplified by conventional RT-PCR; M = 100 bp ladder marker. Gel electrophoresis of RT-PCR products showed the expected 84-bp fragment for GR and 85-bp fragment for MR mRNA in rat heart atria. **(B,C)** Show western blot analyses with anti-GR (lanes 1 and 2) **(B)** and anti-MR (lanes 3 and 4) **(C)** in rat atria show the protein bands with the expected respective molecular weights of 87 kDa for GR and 102 kDa for MR. Each lane represents a tissue sample from a different rat.

Supplementary Figure 3 **(A)** A complete gel of western blot analysis of GR protein bands with rabbit polyclonal anti-GR in rat atria showing protein bands with the expected molecular weights of 87 kDa for GR in addition to several other molecular weight bands in rat atria. **(B,C)** A complete gel of western blot analysis of MR protein bands with mouse monoclonal anti-MR in rat atria and dorsal root ganglia showing protein bands with the expected molecular weights of 102 kDa for MR in addition to several other molecular weight bands in rat atrium **(C)** or DRG **(D)**.

## Abbreviations

BSA: bovine serum albumin
CGRP: calcitonin gene-related peptide
GR: glucocorticoid receptor
HF: heart failure
MR: mineralocorticoid receptor
PBS: phosphate buffer solution
RT-PCR: reverse-transcriptase polymerase chain reaction
SIF cells: small intensely fluorescent cells
TH: tyrosine hydroxylase
VAChT: vesicular acetylcholine transporter.
