# Supplementary figures and images for: Identification of glucocorticoid receptors as potential modulators of parasympathetic and sympathetic neurons within rat intracardiac ganglia
Source: Front Neuroanat. 2022 Sep 23;16:902738. doi: 10.3389/fnana.2022.902738 (PMC9539283; doi:10.3389/fnana.2022.902738)

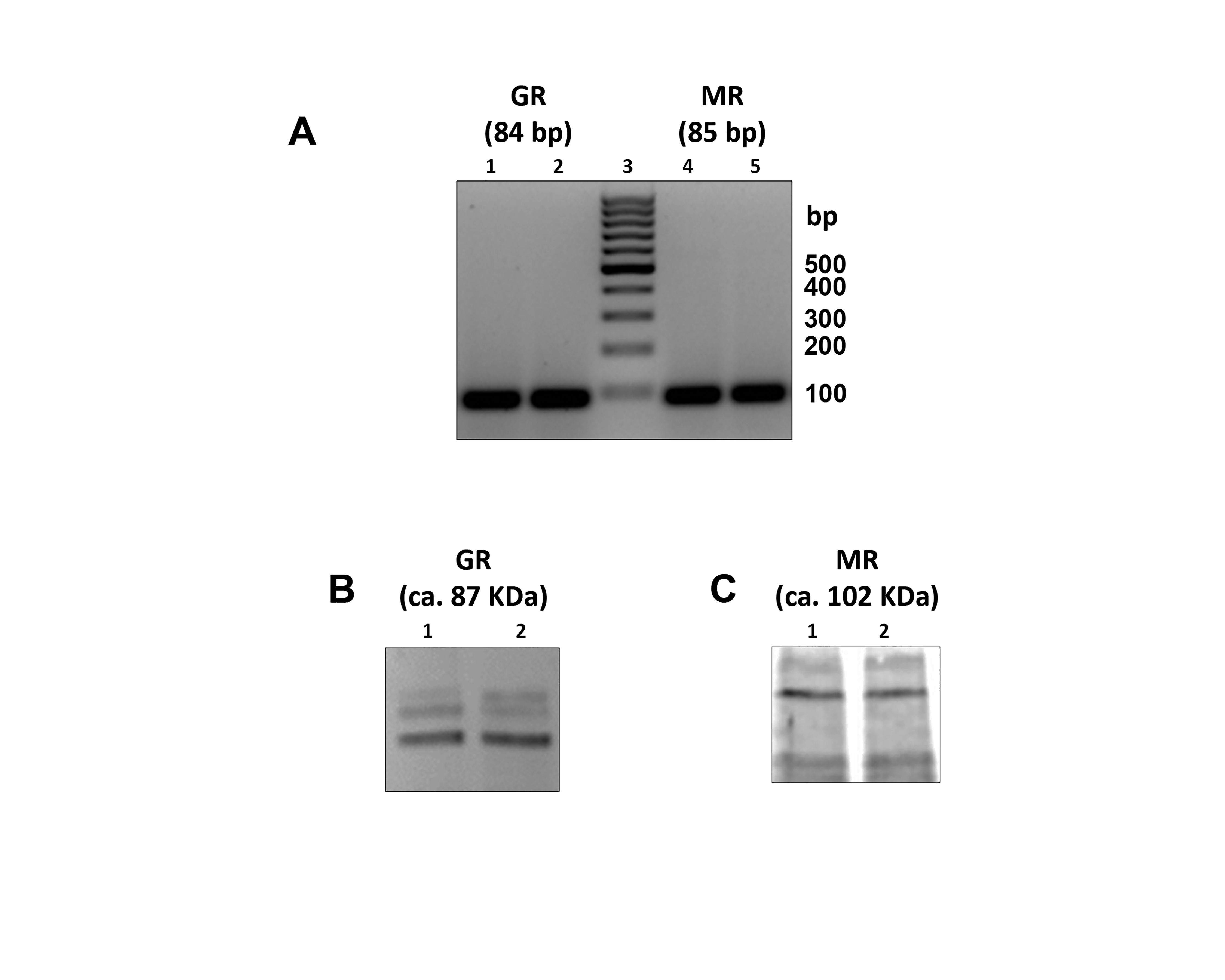

Supplement: Supplementary Figure 1 — Example of quantitative evaluation of immunohistochemical staining within rat atria using the version 1.41 of the image analysis program ImageJ® (http://rsbweb.nih.gov/ij/). The additional use of the plug-in (color deconvolution) allowed the separation of the different color channels each identifying distinct target structures, whose color signal can, thus, be quantitatively evaluated. With the help of ImageJ, the parameter percentage area (% stained area) was calculated. The percentage area was defined as the specific-colored area in relation to the total area of a photographed tissue preparation. [file Image_1.TIF]

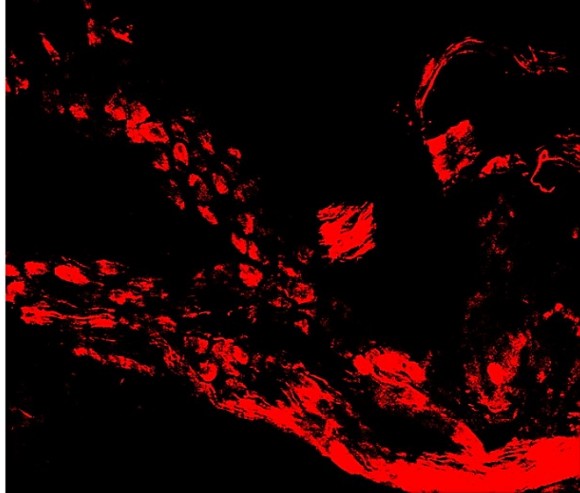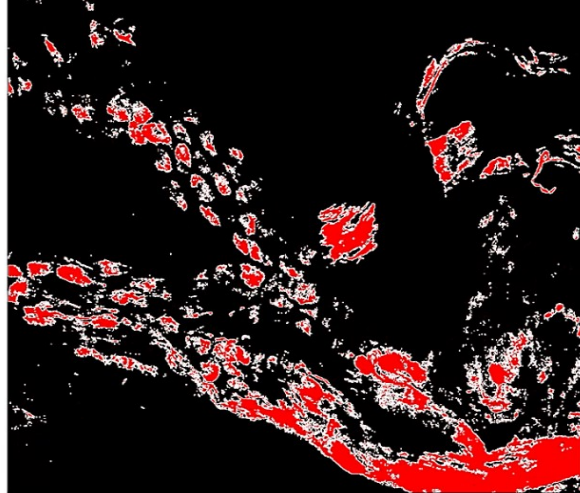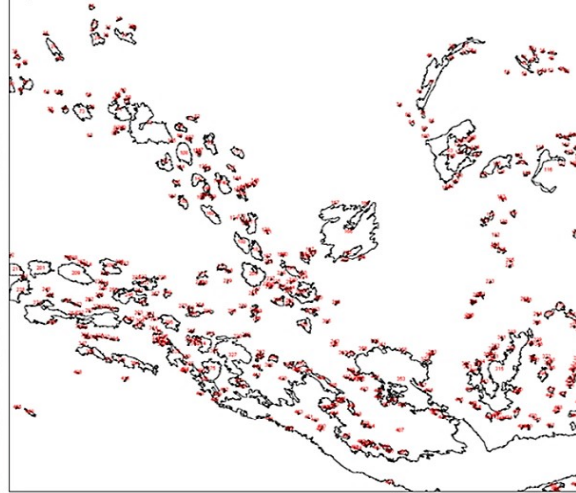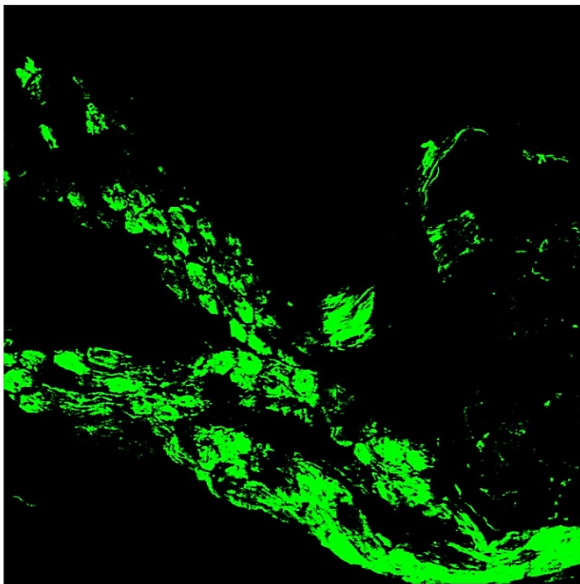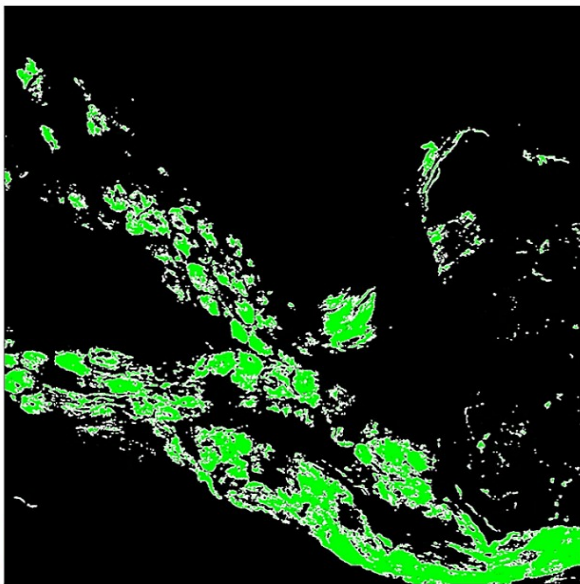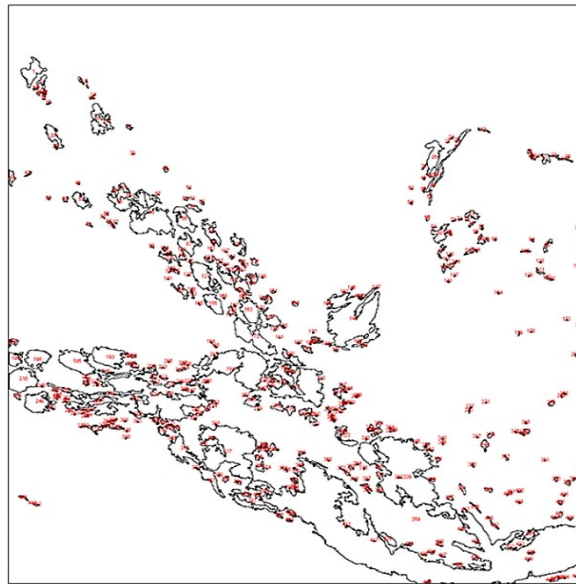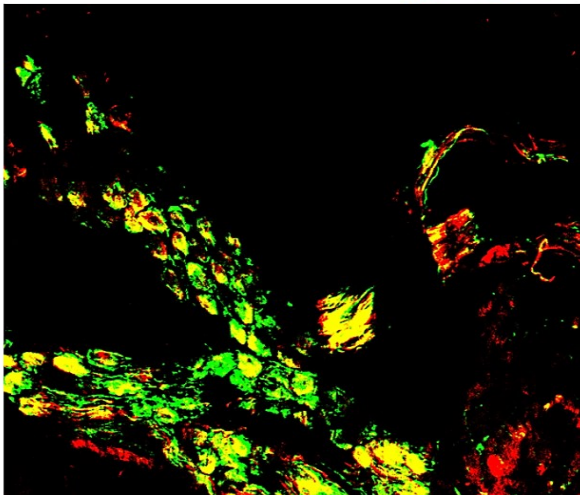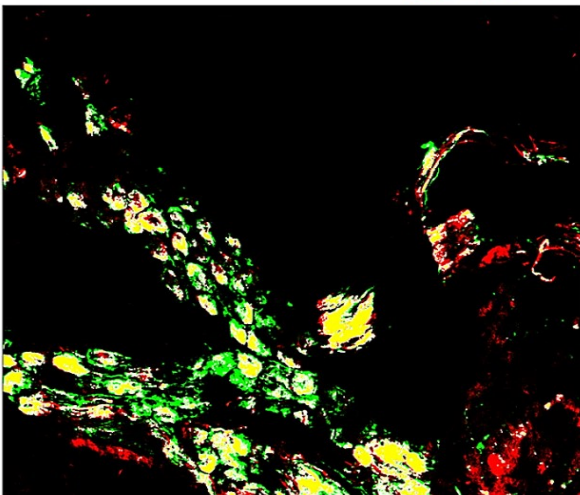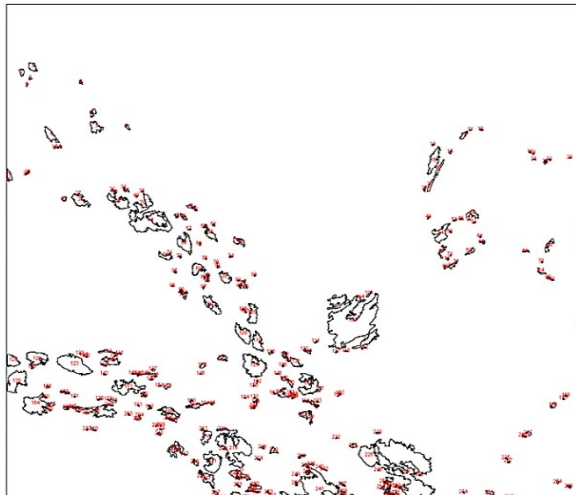

Supplement: Supplementary Figure 2 — Detection of GR and MR mRNA (A) and proteins (B) by conventional RT-PCR and western blots, respectively, in rat atria containing intracardiac ganglia. (A) GR and MR mRNA were amplified by conventional RT-PCR; M = 100 bp ladder marker. Gel electrophoresis of RT-PCR products showed the expected 84-bp fragment for GR and 85-bp fragment for MR mRNA in rat heart atria. (B,C) Show western blot analyses with anti-GR (lanes 1 and 2) (B) and anti-MR (lanes 3 and 4) (C) in rat atria show the protein bands with the expected respective molecular weights of 87 kDa for GR and 102 kDa for MR. Each lane represents a tissue sample from a different rat. [file Data_Sheet_1.PDF]

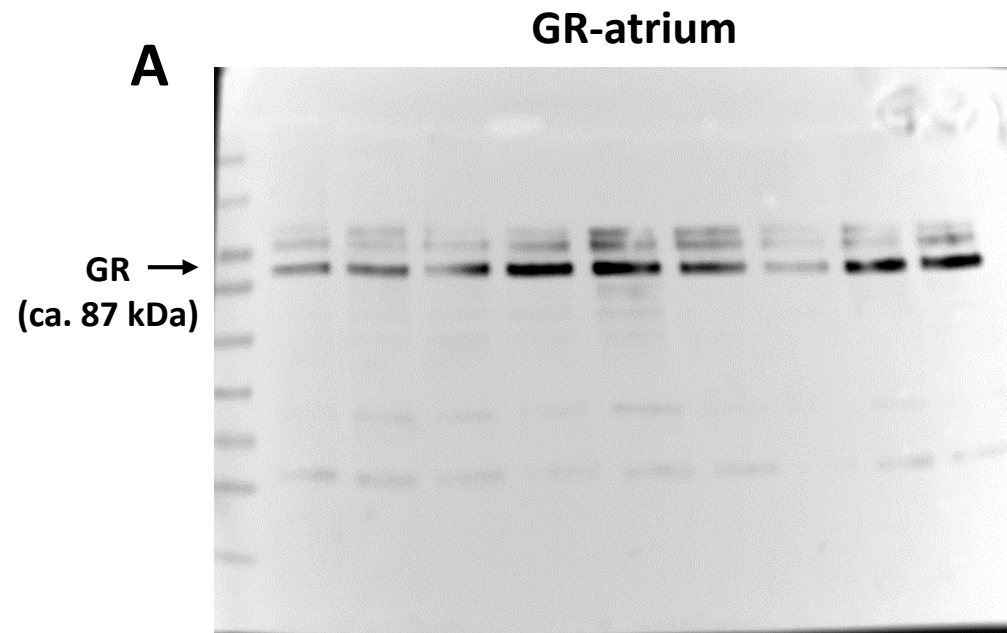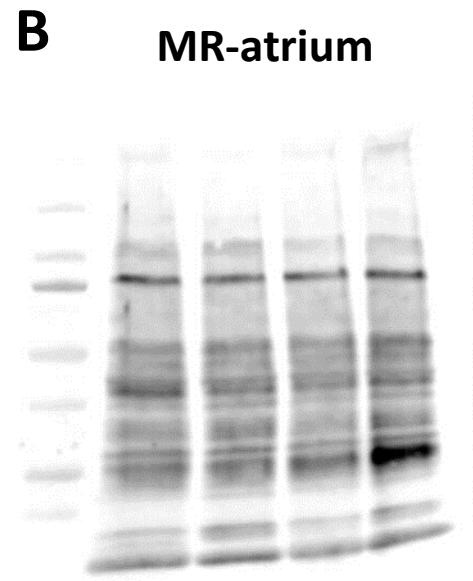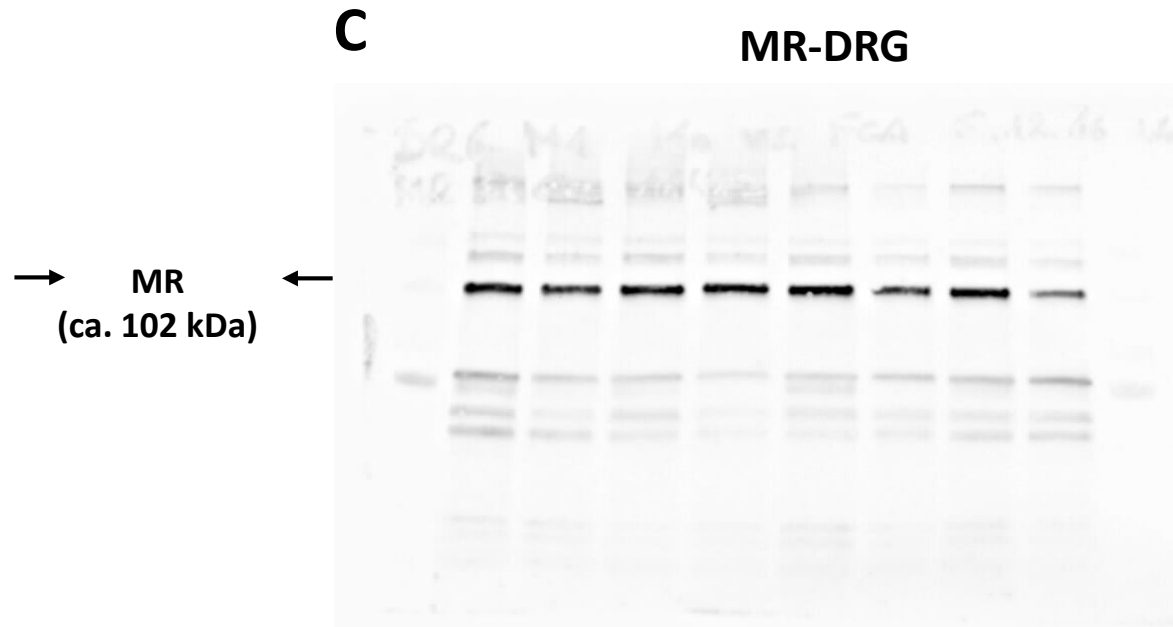

Supplement: Supplementary Figure 3 — (A) A complete gel of western blot analysis of GR protein bands with rabbit polyclonal anti-GR in rat atria showing protein bands with the expected molecular weights of 87 kDa for GR in addition to several other molecular weight bands in rat atria. (B,C) A complete gel of western blot analysis of MR protein bands with mouse monoclonal anti-MR in rat atria and dorsal root ganglia showing protein bands with the expected molecular weights of 102 kDa for MR in addition to several other molecular weight bands in rat atrium (C) or DRG (D). [file Data_Sheet_2.PDF]
